# Development and characteristics of novel sonosensitive liposomes for vincristine bitartrate

**DOI:** 10.1080/10717544.2019.1639845

**Published:** 2019-07-11

**Authors:** Wen Lin, Xiaoxing Ma, Chaopei Zhou, Hong Yang, Yang Yang, Xiangyang Xie, Chunrong Yang, Cuiyan Han

**Affiliations:** a Department of clinical laboratory, Huangshi Love & Health Hospital of Hubei province, Huangshi, China;; b College Pharmacy, Qiqihar Medical University, Qiqihar, China;; c College Pharmacy, Jiamusi University, Jiamusi, China;; d The 4th Affiliated Hospital of Harbin Medical University, Harbin, China;; e Department of Pharmacy, General Hospital of Central Theater of the PLA, Wuhan, China;; f Beijing Institute of Pharmacology and Toxicology, Beijing, China

**Keywords:** Ultrasound sensitivity, sonodynamic effect, sonosensitizer, control release, drug delivery system

## Abstract

The aim of drug delivery is to increase therapeutic efficacy. Externally triggered drug delivery systems enable site-specific and time-controlled drug release. To achieve this goal, our strategy was based on ultrasound-triggered release of an anticancer agent from sonosensitive liposomes (SL). To realize the ultrasound-triggered drug release, a lipophilic sonosensitizer, hematoporphyrin monomethyl ether (HMME) was incorporated into the lipid bilayer of liposomes. Once irradiated by the ultrasound in tumor tissues, the sonodynamic effect generated by HMME could lead to an efficient disruption of the lipid bilayer in the SL. After encapsulating vincristine bitartrate (VIN) as the model drug, the ultrasound-triggered lipid bilayer breakdown can trigger the instant release of VIN, enabling ultrasound-controlled chemotherapy with great specificity. In the *in vitro* and *in vivo* studies, by integrating tumor-specific targeting and stimuli-responsive controlled release into one system, VIN-loaded SL showed excellent antitumor efficacy. The SL could potentially produce viable clinical strategies for improved targeting efficiency of VIN for the treatment of related cancer. More importantly, this report provides an example of controlled release by means of a novel class of ultrasound triggering system.

## Introduction

Over the past few decades, various types of drug delivery systems for cancer therapy have been fabricated from various materials (Zhang et al., 2008). Among these systems, liposomes are often considered as the archetype of many drug delivery vesicles, which were the first ‘nanoscale drug’ approved for clinical use. Upon aggregation into the tumor, the liposomes mainly depend on its passive diffusion to release, which needs a relative long time (Barenholz, 2012). However, passive release is not able to regulate local concentration or amount of drugs in tumor. In addition, the vascular permeability and interior condition of different tumors are largely variable, which will induce unpredictable drug release in the target site (Torchilin, 2005). For these reasons, the liposomes in clinical have only achieved the modest therapeutic index of chemotherapy.

The aim of drug delivery is to increase therapeutic efficacy. The highly localized and burst release of drug in the targeted sites, without unwanted distribution in normal tissues, is the most therapeutically effective way to minimize unfavorable side effects when treating patients (Torchilin , 2012; Khawar et al., 2015; Zhao et al., 2017). To attain this goal, numerous diverse strategies have been proposed to control drug release from liposomes. These include pH-sensitive liposomes, photosensitive liposomes, thermosensitive liposomes, all of which release their contents in response to local physiological or external stimuli (Allen & Cullis, 2013). However, accurate control of drug release in a complex physiological and pathological environment at the appropriate moment with a physiological trigger (e.g. pH) is still a challenge. Thus, it would be preferable to develop an external triggered methodology that is independent of the extracellular tumor microenvironment. Thermosensitive liposomes (ThermoDox^®^) could generate payload release at the phase transition temperature (39–41 °C) by using a mild hyperthermia. However, ThermoDox^®^ is less stable at physiological temperature, exhibiting high leakage of ∼50% within 1 h (Chiu et al., 2005; Li et al., 2013). Overall, it was very difficult to develop a thermosensitive liposome, which in physiological conditions can stably retain drugs in the absence of a heating stimulus but release them in the presence of stimulus (Zhang et al., 2011). Light is quite advantageous due to its noninvasive nature, desirable modulability and high spatial resolution. In recent years, several different groups have attempted to fabricate liposomes responsive to light-triggered photodynamic effects in order to realize controlled release (Qiu & An, 2013). In one example, Lajunen et al. (2016) developed an indocyanine green-decorated liposome that is responsive to light-triggered photodynamic effects to release encapsulated drugs. In this liposome system, indocyanine green, a hydrophobic photosensitizer that can strongly absorb light and generate photodynamic effect (mainly including singlet oxygen and reactive oxygen species), was be inserted into the lipid bilayer of liposomes. Upon light illumination, such photosensitizer-decorated liposomes would be rapidly disrupted due to the photodynamic destruction of the lipid bilayer of the liposome and thus release the loaded drugs. However, the above photosensitive liposomes still need improvements. Light irradiation is typically restricted to superficial tissues, although deep tissues may be reached with the aid of laparoscopy. Thus, the use of light for triggered drug release *in vivo* may be limited.

Unlike visible light, ultrasound is a type of mechanical waves that can penetrate a cancer target buried deep within human tissue. This is a unique advantage compared with electromagnetic modalities such as laser beams in the noninvasive treatment of tumors (Husseini et al., 2014). Recently, sonodynamic therapy (SDT) has emerged as an alternative to the more established photodynamic therapy (PDT) and was considered as a potential anticancer treatment (Rosenthal et al., 2004; Costley et al., 2015). Hematoporphyrin monomethyl ether (HMME), a hydrophobic sonosensitizer/photosensitzer, can strongly absorb ultrasound/light and generate photodynamic/sonodynamic effects (mainly including singlet oxygen and reactive oxygen species) (Tian et al., 2009). To date, HMME, as the first-line anticancer drugs, has been widely used for SDT or PDT in the clinic (Kessel et al., 1995; Misìk & Riesz, 2000). The above reports provided a theoretical foundation for the strategy of ultrasound-triggered drug release.

Herein, a unique type of liposomal drug delivery system is developed, which is responsive to ultrasound-triggered sonodynamic effect and can effectively control the drug release. In this system, HMME was mixed with lipids to prepare sonosensitive liposomes (SL). Upon the exposure to ultrasound, the SL would disrupt rapidly due to the sonodynamic destruction of the lipid bilayers in the liposomes. After loaded with vincristine bitartrate (VIN), a model chemotherapeutic agent for cancer therapy, into the inner aqueous lumen of the SL, the prepared VIN-loaded SL showed an instant VIN release profile upon the exposure to ultrasound. When the target sites are irradiated by ultrasound, VIN will release quickly from the SL, as the membranes of SL are destroyed by the sonodynamic effect. In this work, we described the physicochemical and biological characters of SL based on cancer therapy at the cellular level, and the *in vivo* antitumor efficiencies of VIN-loaded SL were also explored.

## Experimental materials

### Materials

Hydrogenated soy phosphatidylcholine (HSPC) and cholesterol (Chol) were purchased from Lipoid GmbH (Mannheim, Germany). 1,2-distearoyl-sn-glycero-3-phosphoethanolamine-N-methoxy (polyethyleneglycol) (ammonium salt) (DSPE-mPEG_2000_) and 1,2-distearoyl-sn-glycero-3-phosphoethanolamine-N-maleimide (polyethylene glycol) (DSPE-PEG_2000_-Mal) were purchased from Xìan ruixi Biological Technology Co., Ltd (Xìan, China). Hematoporphyrin monomethyl ether (HMME) was purchased from Shanghai Dibo Chemical Technology Co. (Shanghai, China). Sulfate vincristine (VIN) was obtained from Baiyunshan Co. (Guangzhou, China). The other chemicals were all reagent grade and purchased from Millipore Sigma (Darmstadt, Germany), except for special stated.

Human breast adenocarcinoma cells (MCF-7 cells) purchased from the Cell Resource Center of IBMS (Beijing, China), which were maintained in culture medium consisting of Dulbecco’s modified eagle’s medium (DMEM) supplemented with 10% FBS, 100 IU/mL penicillin and 100 mg/mL streptomycin. The cells were maintained in a 37 °C humidified incubator in a 5% CO_2_ atmosphere.

Female Sprague Dawley (SD) rats (200 ± 20 g) and female BALB/c nude mice (weighing 20 ± 2 g) were purchased from the laboratory animal center of Jiamusi University (Jiamusi, China). All animals were handled according to the code of ethics in research, training and testing of drugs as stated by the Animal Care and Use Ethics Committee of Jiamusi University.

## Methods

### Preparation of liposomal nanocarriers

To prepare the normal liposome (NL), a lipid composition (molar ratio) of HSPC (50%), cholesterol (43%) and DSPE-PEG_2000_ (7%) was used. All lipid mixtures were dissolved in chloroform–methanol (3:1, v/v) in a pear-shaped flask and were subsequently evaporated to form a dry film using a rotary evaporator under vacuum. The lipid film was then hydrated using a 300 mM citric acid buffer solution at 50 °C for 30 min. To control for the size, the lipid dispersion was extruded 11 times through 100-nm polycarbonate filters using a mini extruder (Avanti, Canada). The sonosensitive liposomes (SL) were prepared following the same procedures, except the HMME was added to these lipid mixtures at the required molar ratio (1%, 4%, 6% or 8% HMME of total lipid).

VIN was loaded into various liposomal formulations (NL or SL) using the pH gradient method at 1:20 drug/lipids mass ratio as described by our previously report (Li et al., 2016). Briefly, blank liposome suspension prepared as described above was directly added into 300 mM citric acid buffer solution, added into VIN to obtain the drug concentration of 0.5 mg mL^−1^, then adjusted pH of extrinsic phase to 7.5 using 100 mM Na_2_CO_3_ solution and incubated at 55 °C for about 30 min. Finally, the VIN-loaded liposomes (VIN-loaded NL or VIN-loaded SL) were filtration sterilized by 100-nm polycarbonate filter and sub-packed to aseptic vials.

### Quantification of VIN

Determination of VIN was performed by HPLC as described in a previous report (Zhigaltsev et al., 2005), with minor modifications. Briefly, ZORBAX SB-C8 column (4.6 mm × 250 mm, pore size 5 µm) was used, and mixed solution composed of methanol: diethylamine solution (mixture of water and diethylamine (985: 15), adjusted with phosphoric acid to pH 7.5) 70:30 (v/v) was used as mobile phase with the flow rate of 1 mL/min, at 25 °C. Sample injection volume was 20 mL; UV detector was used to detect VIN under 297 nm of the wavelength.

### Evaluation of ultrasound sensitivity

**Choice of acoustic parameters.** VIN-loaded SL (with 6% HMME) was diluted 20-fold with PBS (0.1 M, pH 7.4) and incubated at 37 °C for 10 min and immediately transferred into a HUT-105 ultrasound sonication system (Institute of Biomedical Engineering, Huazhong University of Technology, Wuhan, China). Temperature was measured by inserting a K-type thermocouple probe directly into the solution during ultrasound irradiation. After treating with ultrasound irradiation (1 MHz, 1.5 W cm^−2^ power density, 10-s sonication and with a 10-s pause for a total of 0–130 s), the suspensions were allowed to stand for 10 min and then centrifuged, and the VIN concentration was determined by HPLC as described above.

**Sonosensitizer proportion screening.** The released VIN from the VIN-loaded SL, which contained the HMME at different proportions (1%, 4%, 6% or 8% HMME of the total lipid), was determined following the same procedures, except the acoustic parameters were partially substituted by a total of 110 s.

### Characterization of sonosensitive liposomes

The morphology of VIN-loaded SL was observed by transmission electron microscopy (TEM) (JEM-1010, JEOL Ltd, Tokyo, Japan). The diameter of liposomal nanocarriers was determined in three serial measurements using a photon correlation spectroscopy (Nanophox, Sympatec GmbH, Clausthal-Zellerfeld, Germany). The VIN encapsulation efficiency (EE) of each formulation was determined by HPLC as described above.

### Ultrasound-triggered in vitro release of VIN

Dialysis bags were employed to determine the *in vitro* VIN release profiles of various VIN-loaded liposomal nanocarriers. Briefly, the liposomal VIN sample (1 mL) was transferred to a dialysis bag (molecular weight cutoff of 12,000–14,000 Da) and dialyzed against 20 mL of PBS (0.1 M, pH 7.4) with continuous gentle stirring at 37 °C and treated with or without ultrasound (1 MHz, 1.5 W cm^−2^ power density, 10-s sonication and with a 10-s pause for a total of 110 s). At various time points, 400 μL aliquots were withdrawn from the conical flask for drug analysis, and an equal volume of the medium was added. The leakage of VIN was determined via HPLC as described above.

### Storage stability

The long-term storage stability of VIN-loaded SL was evaluated. The liposomal nanocarriers were stored at 4 °C in closed amber vials without any other precautions, and samples were periodically removed for routine analysis. The stability parameters, such as diameter, PDI, EE and ultrasound-triggered release rates, were assessed every month for 3 months.

### Cellular viability

The cytotoxicities of various VIN-loaded liposomal formulations and free VIN on MCF-7 cells were measured via the MTT assay. Briefly, the cells were seeded on a 96-well plate at a density of approximately 4000 cells/well. After incubation for 24 h (37 °C, 5% CO_2_), the cells were treated with various VIN-loaded liposomal formulations or free VIN at a range of concentrations. In the liposomal formulation groups (VIN-loaded NL and VIN-loaded SL), those samples were pretreated with or without ultrasound, as mentioned above. Then, after 72 h, MTT solutions (20 μL, 5 mg mL^−1^) were applied to the wells. After incubation for 4 h, the cell viability (%) was calculated based on the collected UV data via a Model 680 plate reader (Bio-Rad, USA).

### Pharmacokinetic studies

The pharmacokinetic profiles of VIN-loaded NL and VIN-loaded SL were measured in SD rats with a single dose of 1.0 mg kg^−1^ VIN (i.v. via tail vein, diluted to 0.5 mL by physiological saline). Blood was sampled from the retro-orbital sinus at different time points. To prepare samples for analysis, 0.1 mL internal standard (I.S.) solution (vinblastine sulfate, 40 ng mL^−1^) was added to 100 µL plasma sample in a 1.5-mL test tube. The sample mixture was deproteinized with 0.8 mL of methanol and vortex-mixed for approximately 1 min, and the precipitate was removed by centrifugation at 12,000 rpm (revolutions per minute) for 10 min. Then, 800 µL of supernatant was transferred to another clean test tube and evaporated to dryness at 37 °C with a CentriVap Concentrator. The dry residue was reconstituted in 100 µL of the mobile phase, vortex-mixed and centrifuged at 12,000 rpm for another 10 min; 20 μL of the clean supernatant was injected into the HPLC for analysis. The analysis was performed using Venusil MP-C18 (4.6 mm × 250 mm, pore size 5 µm) HPLC column at 20 °C. The mobile phase was a methanol–acetonitrile–phosphate buffer (pH 7, 23: 52: 25, vol. %), at a flow rate of 1.0 mL min^−1^. VIN detection was performed using UV detector at a wavelength of 220 nm.

### Establishment of tumor model

A xenograft tumor model was produced via subcutaneous injection of MCF-7 cells as described in previous report (Xie et al., 2015). Briefly, MCF-7 cells in exponential phase of growth were digested and resuspended at a final concentration of 5 × 10^7^/mL in serum-free RPMI-1640 culture medium. Several nude mice were subcutaneously inoculated with 1 × 10^7^ MCF-7 cells in left axilla. Upon reaching tumor size of about 5 mm in diameter after 10-day growth, we began to prepare for the following experiments.

### Tissue biodistribution

Tissue biodistribution of VIN-loaded SL was performed on nude mice-bearing tumor xenografts, and compared with that of VIN-loaded NL and free VIN. The mice were randomly divided into four groups and were treated as follows via intravenously administration: free VIN; VIN-loaded NL; VIN-loaded SL (with ultrasound) and VIN-loaded SL (without ultrasound) at a dose of 4.0 mg kg^−1^ VIN, respectively. Thirty minutes after the administration, the tumor-xenografted mice treated with VIN-loaded SL were anesthetized, and the surface of the tumor sites was covered with 1.0-cm-thick gel interfaces (EcoGel 100 Imaging Ultrasound Gel, Eco-Med Pharmaceutical Inc., Mississauga, Ontario, Canada). Then, each tumor site was treated with a HUT-105 ultrasound sonication system (Institute of Biomedical Engineering, Huazhong University of Technology, Wuhan, China) with a sonication area of 0.8 cm^2^ and ultrasound of 1 MHz, 1.5 W cm^−2^, a 10-s sonication with 10-s pause for a total of 110 s. Meanwhile, other groups that were not subjected to ultrasound were used as controls. One hour after the i.v. injection, three mice of each group were sacrificed with heart, liver, spleen, lung, kidney and tumor collected and stored at –20 °C until analysis. For VIN concentration detection, the samples were homogenized in threefold volumes of distilled water with the homogenate pretreated via a protein precipitation procedure using vinblastine as the I.S. and the supernatant following centrifugation was subjected to LC/MS/MS analysis as described above.

### *In vivo* antitumor efficacy

The MCF-7 tumor-xenografted mice were injected in their tail vein with 5% glucose (control), 1.0 mg kg^−1^ of free VIN, VIN-loaded NL and VIN-loaded SL on the 6th, 9th, 12th and 15th day. Thirty minutes after the administration, the group cured with VIN-loaded SL was irradiated with or without ultrasound, as described above. The tumor volumes were measured. The estimated tumor volume was calculated using the formula: volume (mm^3^) = (length × width^2^)/2. The survival time was calculated from day 0 (tumor inoculation) to the day of death. Kaplan–Meier survival curves were plotted for each group. Meanwhile, the body weight of each mouse was measured daily.

### Statistical analysis

The data are presented as the means ± standard deviation (SD). The difference between any two groups was determined via ANOVA. The *p* value less than .05 (*p* < .05) indicated significance.

## Results and discussion

### Ultrasound triggering potential of sonosensitive liposomes

**Figure 1.**
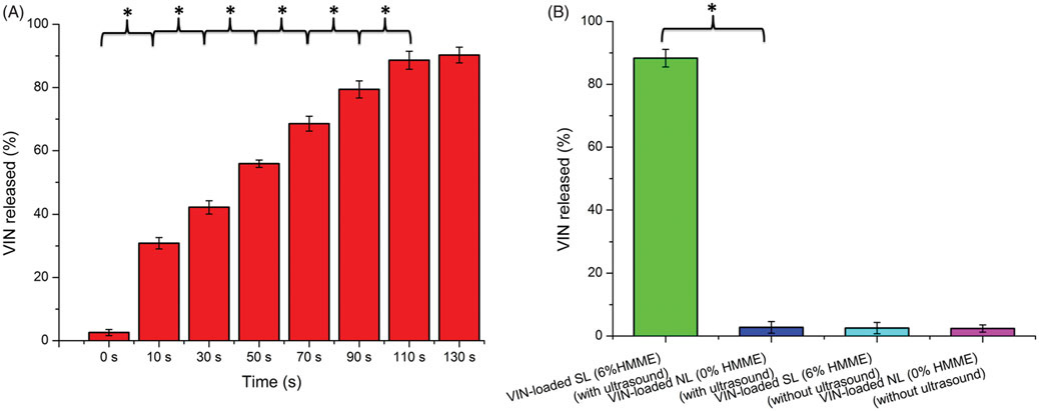
In vitro release of VIN-loaded SL (6% HMME) with different ultrasound irradiated time in PBS (0.1 M, pH 7.4) at 37 °C (A). In vitro release of VIN-loaded SL and VIN-loaded NL with or without ultrasound in PBS (0.1 M, pH 7.4) at 37 °C (B). The data are presented as the means ± SD (*n* = 3). * indicates *p* < .05.

Recently, many groups have reported that HMME could generate a large amount of ROS when irradiated by light for PDT or by ultrasound for SDT. Ultrasound has many advantages over light, especially the deep tissue penetration ability. Therefore, in this study, ultrasound was used as an external stimulus to irradiate liposomes containing HMME for controlled drug release. As illustrated in Figure 1(A), with an increase in ultrasound irradiation time (0–110 s), there was a notable improvement in the degree of drug release. When the time was 110 s (10-s sonication with 10-s pause), approximately 88.62% ± 2.84% of the VINs were released from the SL. With further increases in the time to 130 s, there was no remarkable difference in releasing degree compared to that of 110 s (*p* > .05). Thus, 110 s (10-s sonication with 10-s pause) of ultrasound stimulus was used in further experiments.

To confirm the specific requirement of sono-activated HMME for the sono-triggering and VIN release, VIN-loaded NL (with 0% HMME) and VIN-loaded SL (with 6% HMME) were tested under identical ultrasound conditions (1 MHz, 1.5 W cm^−2^ power density, a 10-s sonication and with a 10-s pause for a total of 110 s). As shown in Figure 1(B), without ultrasound stimulus, the VIN-loaded NL (with 0% HMME) or VIN-loaded SL (with 6% HMME) exhibited minimal payload leakage in the medium with a release less than 3% after the incubation at 37 °C for 10 min. In contrast, during the incubation of 10 min, VIN-loaded SL (with 6% HMME) showed a substantial amount (over 85% of VIN) release after treatment with ultrasound irradiation, whereas only a little of the VIN (below 3% of VIN) was released from VIN-loaded NL (with 0% HMME). This confirmed that HMME was essential for sono-triggering. In addition, in the absence of ultrasound stimulus, the solution temperature remained constant at 37 °C and there was a little of the VIN release. When the VIN-loaded SL (with 6% HMME) was exposed to ultrasound irradiation, VIN release from SL occurred without any significant temperature increase in the solution. This result indicates a difference from conventional stimulus-triggered liposomal release mechanisms that rely on heating to trigger phase transitions.

Since the content of HMME in liposomes was a key factor that influenced the drug release efficiency of SL, the release of VIN from different liposomal formulations with various HMME proportions (1%, 4%, 6% and 8% HMME of total lipid) were evaluated to guide the formulation optimizing process. As exhibited in Figure S1, when the HMME proportion reached 4% in the SL, there was a substantial amount of drug release after treating with ultrasound stimulus. The results indicated that when the proportion of HMME in the SL was lower, HMME could not generate a sufficient sonodynamic effect to effectively disrupt the lipid bilayer structures of the liposomes (under ultrasound stimulus), and thus, the release efficiency of SL was not ideal. When the HMME proportion reached 6%, upon ultrasound stimulus, over 85% of the VIN was immediately released from the SL. With further increase of HMME ratio to 8%, there was no remarkable increase of drug release compared with the 6% ratio formulation (*p* > .05). This result indicated that the acoustic parameters (1 MHz, 1.5 W cm^−2^, a 10-s sonication with a 10-s pause for a total of 110 s) might be insufficient to trigger all of the HMMEs (8%). These results demonstrated that the 6% HMME proportion in the SL was sufficient to generate a sonodynamic effect under current acoustic conditions to trigger a burst release of VIN loaded in the liposomes. Therefore, a molar proportion of 6% for HMME was chosen for the subsequent studies.

### Characterization and in vitro release of sonosensitive liposomes

**Figure 2.**
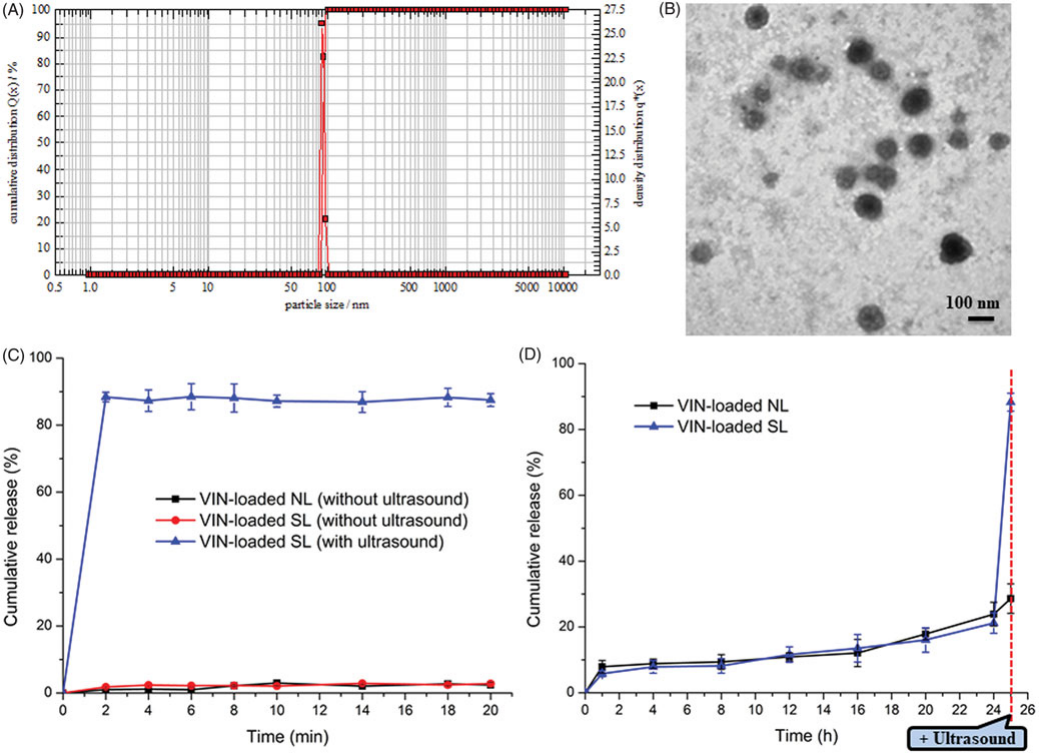
Particle size distribution of VIN-loaded SL (A). Morphological appearance of VIN-loaded SL based on TEM (B). In vitro release of VIN from various liposomal formulations in PBS (0.1 M, pH 7.4) at 37 °C (C). In vitro release of VIN-loaded SL and VIN-loaded NL with ultrasound after a 24-h incubation in PBS (0.1 M, pH 7.4) at 37 °C (D). The data are presented as the means ± SD (*n* = 3).formulations in PBS (0.1 M, pH 7.4) at 37 °C (C). In vitro release of VIN-loaded SL and VIN-loaded NL with ultrasound after a 24-h incubation in PBS (0.1 M, pH 7.4) at 37 °C (D). The data are presented as the means ± SD (*n* = 3).

The physicochemical properties of the two diverse liposomal nanocarriers are presented in Table 1. The VIN encapsulation efficiency (EE) of the two liposomes was more than 90%. The results indicated that the lipids mixed with HMME did not influence the final EE. For nanocarriers, particle size would be a precondition and a crucial factor that determined the fate of nanocarriers both *in vivo* and *in vitro*. After EE study, the particle sizes of these liposomal nanocarriers were further analyzed by a laser particle analyzer (Figure 2(A)). The sizes of the VIN-loaded SL and VIN-loaded NL were 106.84 ± 1.37 nm and 105.51 ± 1.18 nm, respectively. We concluded that the size of the liposomes was not significantly affected by the HMME decoration. As shown in Figure 2(B), TEM images of VIN-loaded SL demonstrated that the particle sizes were similar to those determined by a laser particle analyzer (Figure 2(A)).

**Table 1.** Characteristics of the liposomal nanocarriers.

Sample ID	Particle size (nm)	PDI	EE (%)	Drug loading (%)
VIN-loaded NL	105.51 ± 1.18	0.061 ± 0.021	93.03 ± 0.92	3.63 ± 0.03
VIN-loaded SL	106.84 ± 1.37	0.058 ± 0.018	91.82 ± 1.03	3.58 ± 0.04

The data are expressed as the mean ± SD for three different preparations (*n* = 3).

The *in vitro* release of VIN from various liposomal nanocarriers by sonication is depicted in Figure 2(C). The data revealed the release of VIN from SL exhibited an ultrasound-dependent characteristic. When VIN-loaded SL was exposed to ultrasound, liposomes were effectively disrupted by the sonodynamic effect, leading to the burst release of VIN. In contrast, Figure 2(D) demonstrated that there was minimal release (less than 25%) from VIN-loaded SL, throughout a 24-h incubation in medium without ultrasound stimulus. However, when these samples were subjected to ultrasound irradiations at the 25th hour, burst release (around 90%) occurred, suggesting the intended activation of sonosensitizer. It was also illustrated that SL could be present stable until coming into the contact with ultrasound. Therefore, it was anticipated that only a little of drug would be released into circulation from these ultrasound-responsive nanocarriers before the exposed to ultrasound.

The stability tests showed that VIN-loaded SL was physically and chemically stable at 4 °C up to 3 months. As shown in Table S1, no significant change in EE of VIN-loaded SL was observed during the stability study. The size of VIN-loaded SL remained close to 108 nm, together with a low polydisperity index near to 0.07, indicating a small and monodisperse population of nanoparticles. The ultrasound-triggered VIN release degree from VIN-loaded SL remained almost unchanged during storage. As shown in Figure S2, upon ultrasound irradiation, over 87% of the VIN was released from VIN-loaded SL. The results indicated that the VIN-loaded SL performed consistently in functional assays as being stable in the absence of ultrasound. The stable VIN-loaded SL would thus be favorable for further applications in the clinic.

### Cytotoxicity

The cytotoxicities of free VIN and various VIN-loaded liposomal formulations with or without ultrasound irradiation were evaluated after incubated with the aforementioned cells for 72 h. As shown in Figure S3, without ultrasound irradiation, free VIN could result in the greatest antiproliferative effects on MCF-7 cells. It could be concluded from this result that free drugs could be quickly transported into cells by passive diffusion with a high concentration gradient under *in vitro* conditions, whereas drug-loaded liposomal formulations without ultrasound are needed to undergo the drug release process (Figure 2(C,D)) after entering the intracellular region. Thus, free VIN exhibited a stronger inhibitory effect on the proliferation of monolayer MCF-7 cells compared with various liposomal formulations without ultrasound irradiation. Following the treatment with ultrasound irradiation, compared with VIN-loaded NL regardless of ultrasound exposure, VIN-loaded SL (with ultrasound) displayed a significant improvement in cytotoxicity, to a similar level as the free VIN did, suggesting that VIN was poured out of SL by ultrasound stimulus. These results indicated that the VIN-loaded SL’s antiproliferative activities were depended on the presence of ultrasound irradiation. However, VIN-loaded NL was not affected by ultrasound treatment. These findings are in accordance with the *in vitro* release results as displayed in Figure 1(B). Taken together, the results from the cell studies have roughly validated several points of our design, but this is not enough. To verify the actual target and release abilities of the drug delivery strategy, *in vivo* studies are necessary.

**Figure 3.**
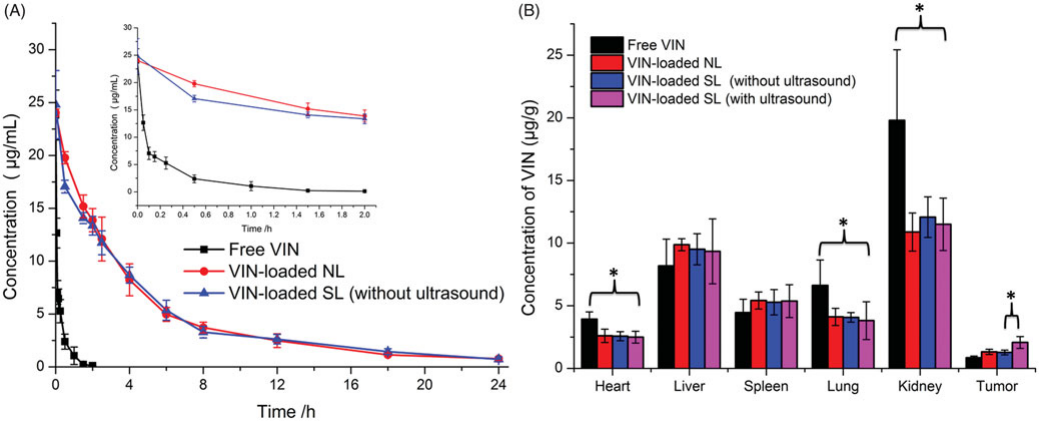
Plasma VIN concentration-time profiles after i.v. injection of different formulations in rat (*n* = 3) (A). Concentration of VIN in the major organs 0.5 h after i.v. injection (B). The data are presented as the means ± SD (*n* = 3). * indicates *p* < .05.

### Pharmacokinetic and tumor targeting property studies

The plasma concentration-time profiles and pharmacokinetic parameters of VIN after i.v. administration of free VIN and VIN-loaded liposomal nanocarriers without ultrasound are shown in Figure 3(A) and Table S2, respectively. It was found that VIN-loaded NL and VIN-loaded SL (without ultrasound) showed similar blood concentration-time curves. Both the sonosensitive liposomes and normal liposomes showed initial high blood circulating levels, while free VIN was quickly cleared from the systemic circulation. Furthermore, VIN-loaded liposomal nanocarriers (VIN-loaded NL and VIN-loaded SL) demonstrated a significantly slower clearance rate (CL) and higher AUC when compared with free VIN. As expected, no significant difference between CL and AUC was observed between two liposomal nanocarriers. This suggests that the mixing of HMME in the lipids of liposomes did not impair the long-circulation characteristics of DSPE-PEG_2000_.

The selective distribution of drug-loaded nanocarriers in tumors could potentially benefit the anti-tumor efficiencies of chemotherapy *in vivo*. To verify this, concentrations of VIN in solid tumors were evaluated in MCF-7 xenograft-bearing nude mice following i.v. administration of free VIN, VIN-loaded NL and VIN-loaded SL with or without ultrasound, respectively. As shown in Figure 3(B), the concentrations of VIN in the tumors of VIN-loaded SL (with ultrasound) were 2.5-fold higher than that of free VIN, 1.58-fold higher than that of VIN-loaded NL and 1.60-fold higher than that of VIN-loaded SL (without ultrasound). These results indicated that VIN-loaded SL (with ultrasound) achieved a higher VIN concentration in tumors than that of VIN-loaded NL and free VIN, probably owing to the ultrasound-triggered release at tumor sites. In other words, VIN-loaded SL combined with ultrasound could realize the targeted drug release at tumor sites. However, VIN-loaded SL (with ultrasound) exhibited a similar tissue distribution profile as the VIN-loaded NL and VIN-loaded SL (without ultrasound) did in the non-targeted tissues. In the free VIN group, a significantly higher level of VINs was observed accumulating in the liver, heart, spleen and kidney.

Taken together, VIN-loaded SL (with ultrasound) possesses desirable pharmacokinetic and tumor-distribution profiles, making it suitable for an *in vivo* tumor targeting drug delivery via systemic administration.

### *In vivo* antitumor activity

**Figure 4.**
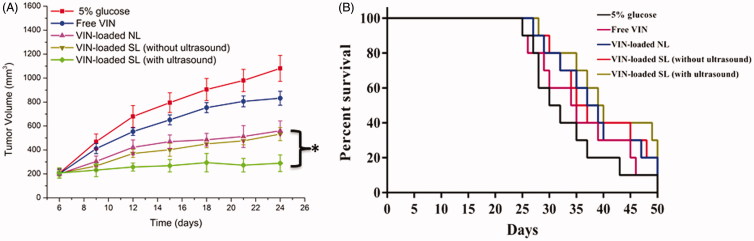
Antitumor activity (A) and survival curve (B) in MCF-7 tumor-bearing mice after treatments with 5% glucose, free VIN and varying formulations carrying VIN. The data are presented as the means ± SD (*n* = 10). * indicates *p* < .05.

To determine whether VIN-loaded SL (with ultrasound) possesses antitumor activity *in vivo*, the effects of free VIN, VIN-loaded NL, VIN-loaded SL (without ultrasound) and VIN-loaded SL (with ultrasound) on tumor growth inhibition in animals were investigated. As shown in Figure 4(A), the tumor volumes of mice in control group rapidly increased over 18 d, whereas that of mice administered with free VIN or various liposomal formulations displayed various tumor inhibition effects. Compared with VIN-loaded NL, VIN-loaded SL (without ultrasound) exhibited similar tumor inhibition effects. As expected, the maximal reduction in tumor growth was found in the group treated with VIN-loaded SL (with ultrasound). In addition, the median survival time of the groups treated with 5% glucose, free VIN, VIN-loaded NL, VIN-loaded SL (without ultrasound) and VIN-loaded SL (with ultrasound) were 31, 35, 38, 37 and 40 d, respectively (Figure 4(B)). By statistical analysis, the VIN-loaded SL (with ultrasound) group has the longest median survival time in the MCF-7 models (*p* < .05). These results were correlated with the abovementioned data revealing the merit of VIN-loaded SL (with ultrasound) over the other liposomal nanocarriers we tested in cytotoxicity induction *in vitro* (Figure S3) and tumor-specific distribution *in vivo* (Figure 3(B)). And it indicated the efficacy of the ultrasound activation on VIN-loaded SL.

The changes in the body weights of the animals were recorded as a safety evaluation index. As shown in Figure S4, there were no significant changes in the body weights of mice treated with VIN-loaded liposomal nanocarriers during the experimental period (*p* > .05). These results suggest that there is negligible acute or severe toxicity related to the present treatments at the tested dose. However, more than 15% weight loss was detected in the free VIN group at the end of the experiment. The weight loss of the free VIN group was likely due to the non-targeted effects of drugs and tumor cachexia.

## Conclusions

A novel sonosensitive liposomal nanocarrier (SL)-contained sonosensitizer HMME was successfully developed in this study. The SL accumulates in the tumor site due to the EPR effects and then is disrupted under certain ultrasound irradiation due to the sonodynamic effect. As a result, most of the drug loaded inside the liposomal nanocarriers would release suddenly when triggered by the ultrasound. In the *in vitro* and *in vivo* studies, combining with ultrasound treatment, VIN-loaded SL showed excellent antitumor efficacies to the target tumors. These research data indicated that SL is expected to be a promising delivery system of anticancer drugs for oncotherapy. In addition, SL might be seen as an alternative to sonodynamic therapy, in which ultrasound is used to generate reactive oxygen species locally at the tumor site that causes cancer cell death but cannot induce the delivery of drugs. To improve the targeting ability of SL, tumor-specific targeting ligands could be added onto the surfaces of the reported nanocarriers here. In future studies, we will continue to improve on the *in vivo* study by including survival analysis and by further exploring the application of SL in oncotherapy.

## Supplementary Material

Supplemental Material
