# Supplementary material for: Development and characteristics of novel sonosensitive liposomes for vincristine bitartrate
Source: Drug Deliv. 2019 Jul 11;26(1):724–31. doi: 10.1080/10717544.2019.1639845 (PMC6691763; doi:10.1080/10717544.2019.1639845)
Supplement: Supplemental Material [file IDRD_A_1639845_SM9330.zip › Supporting_Information_R.docx]

**Supporting Information**

**
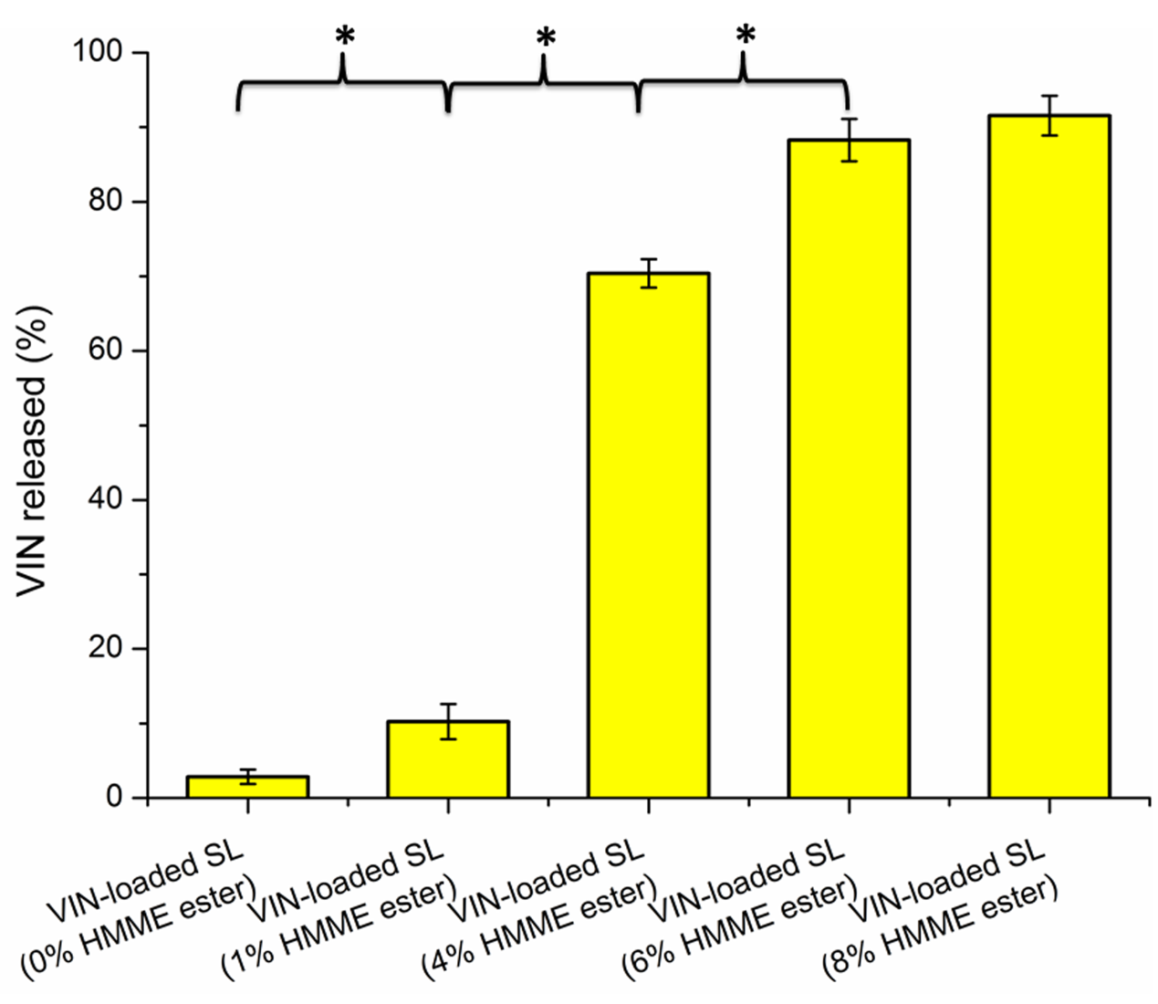
**

**Figure S1.**Effect ofvariousHMMEproportions in formulations on the efficiency of ultrasound-triggered VIN releasein PBS (0.1 M, pH 7.4)at 37 °C.The data are presented as the means ± SD (n = 3).

**

**

**Figure S2.** *In vitro* release of VIN in PBS (0.1 M, pH 7.4) at 37 °C upon ultrasound irradiation, these VIN-loaded SL were stored 0-3 months at 4°C. The data are presented as the means ± SD (n = 3).

**
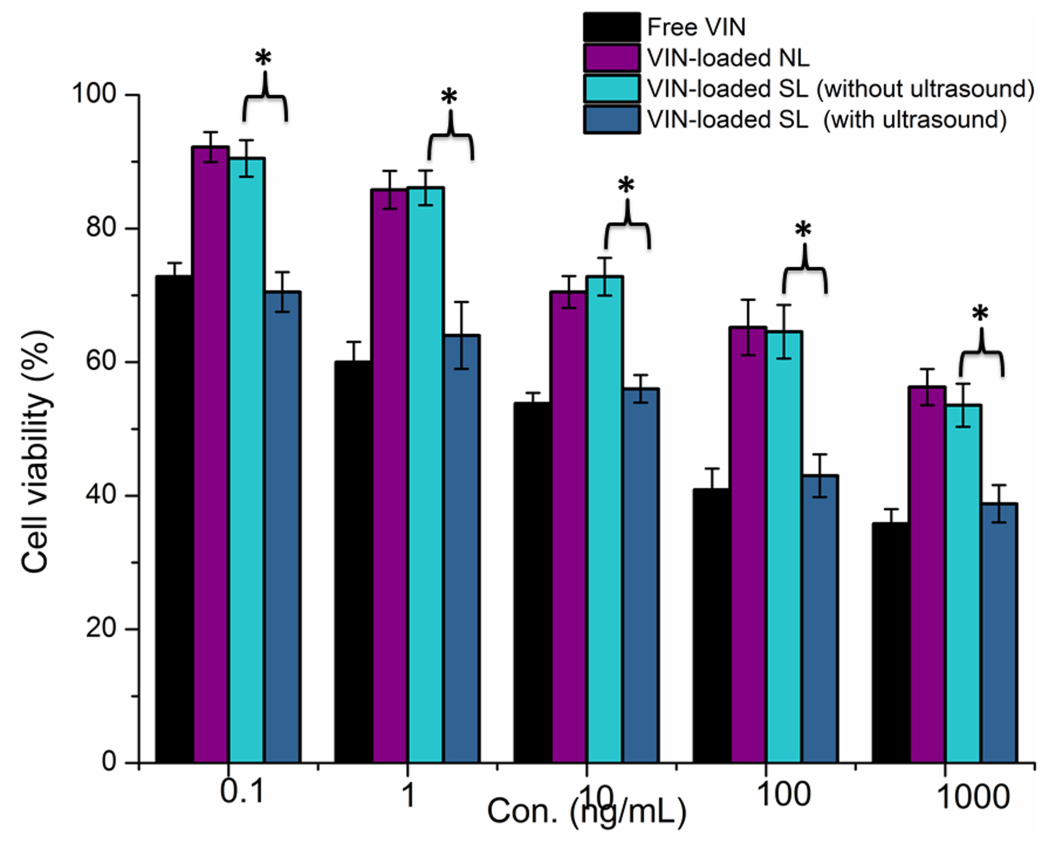
**

**Figure S3.**The cytotoxicity of free VIN and VIN-loaded various liposomalformulations. The data are presented as the means ± SD (n = 3). * indicates P< 0.05.

**
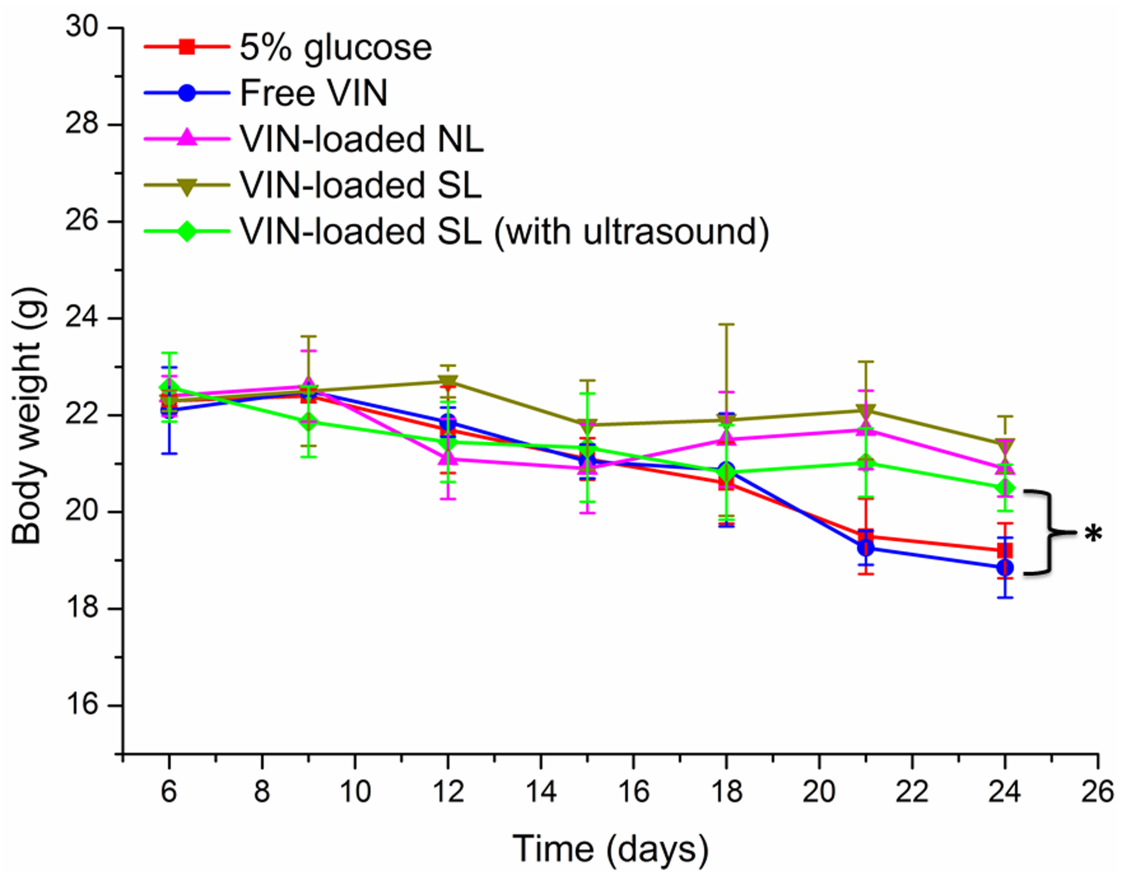
**

**FigureS4.**Body weight changesin MCF-7 tumor-bearing mice after treatments with 5% glucose, free VIN and varying formulations carrying VIN. The data are presented as the means ± SD (n = 10). * indicates P< 0.05.

**Table S1**. Stability data of VIN-loaded SL at 4°C

| Time (month) | Particle size (nm) | PDI | EE (%) |
| --- | --- | --- | --- |
| 0 | 106.84 ± 1.37 | 0.058 ± 0.018 | 91.82 ± 1.03 |
| 1 | 107.13 ± 1.01 | 0.062 ± 0.017 | 91.54 ± 0.81 |
| 2 | 106.95 ± 0.87 | 0.059 ± 0.018 | 91.22 ± 1.06 |
| 3 | 107.66 ± 1.26 | 0.067 ± 0.021 | 91.19 ± 0.73 |

The data are expressed as the mean ± SD for three different preparations (n = 3).
