# Supplementary figures and images for: Development and characteristics of novel sonosensitive liposomes for vincristine bitartrate
Source: Drug Deliv. 2019 Jul 11;26(1):724–31. doi: 10.1080/10717544.2019.1639845 (PMC6691763; doi:10.1080/10717544.2019.1639845)

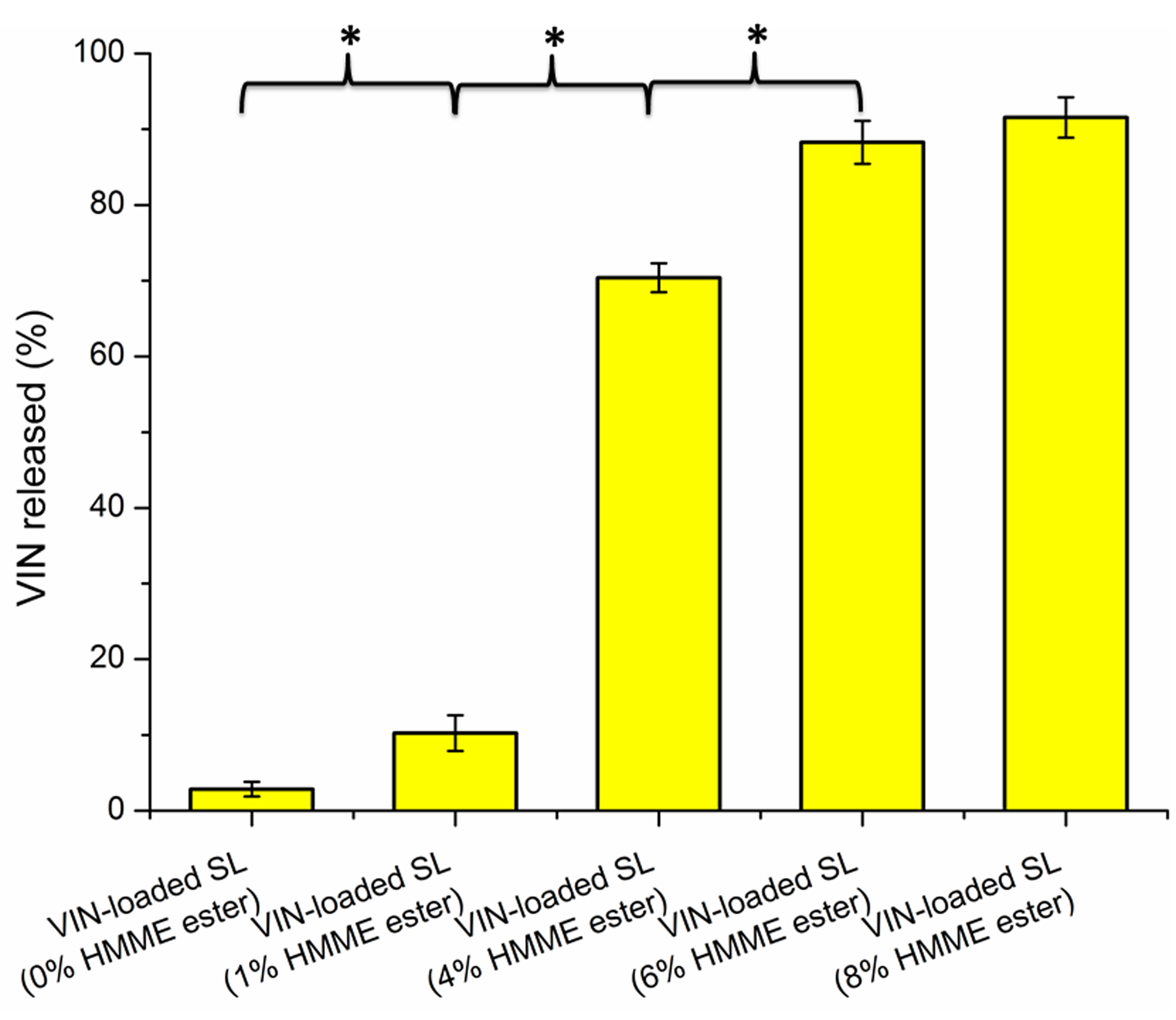

Supplement: Supplemental Material [file IDRD_A_1639845_SM9330.zip › Fig._S1.tif]

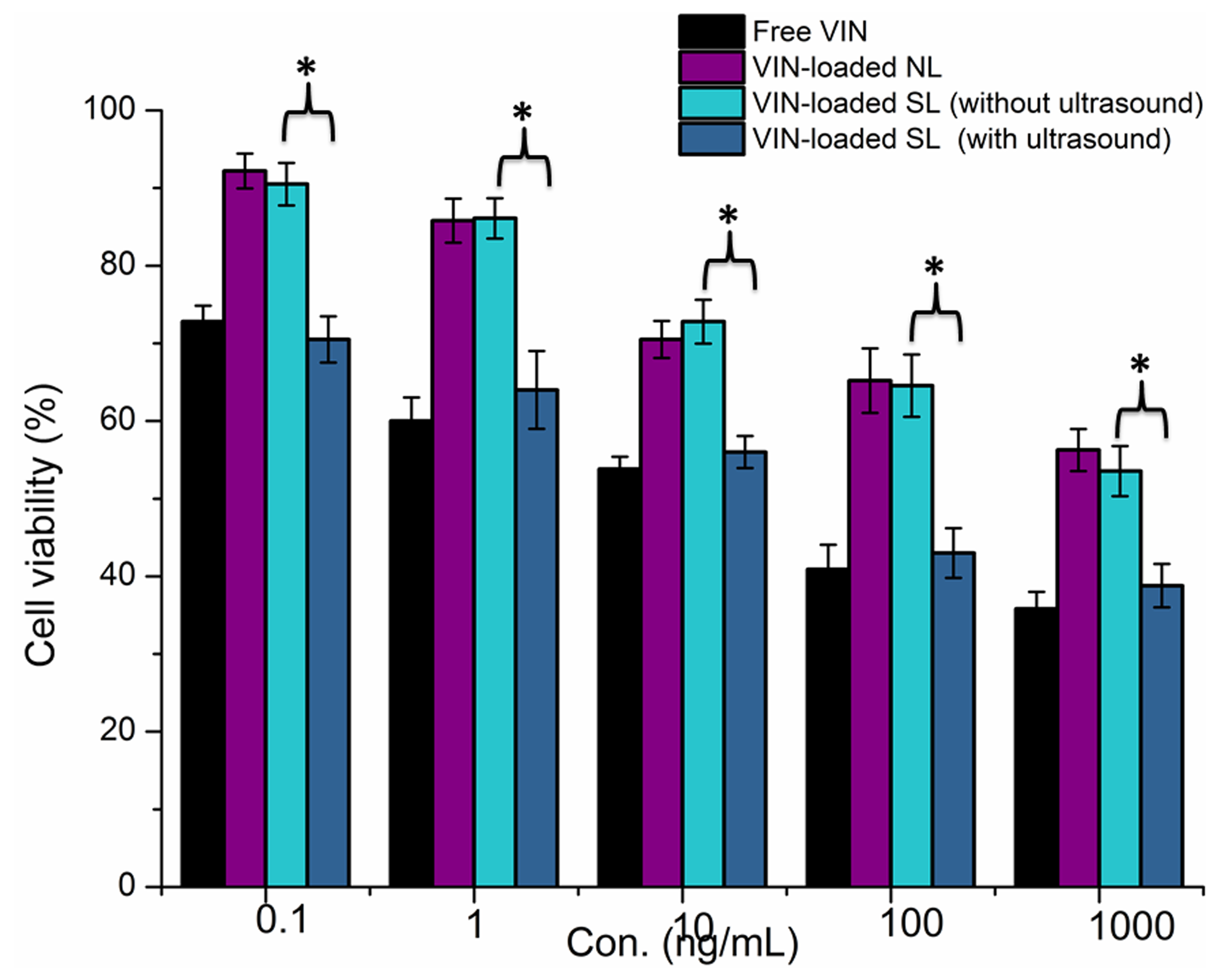

Supplement: Supplemental Material [file IDRD_A_1639845_SM9330.zip › Fig._S3.tif]

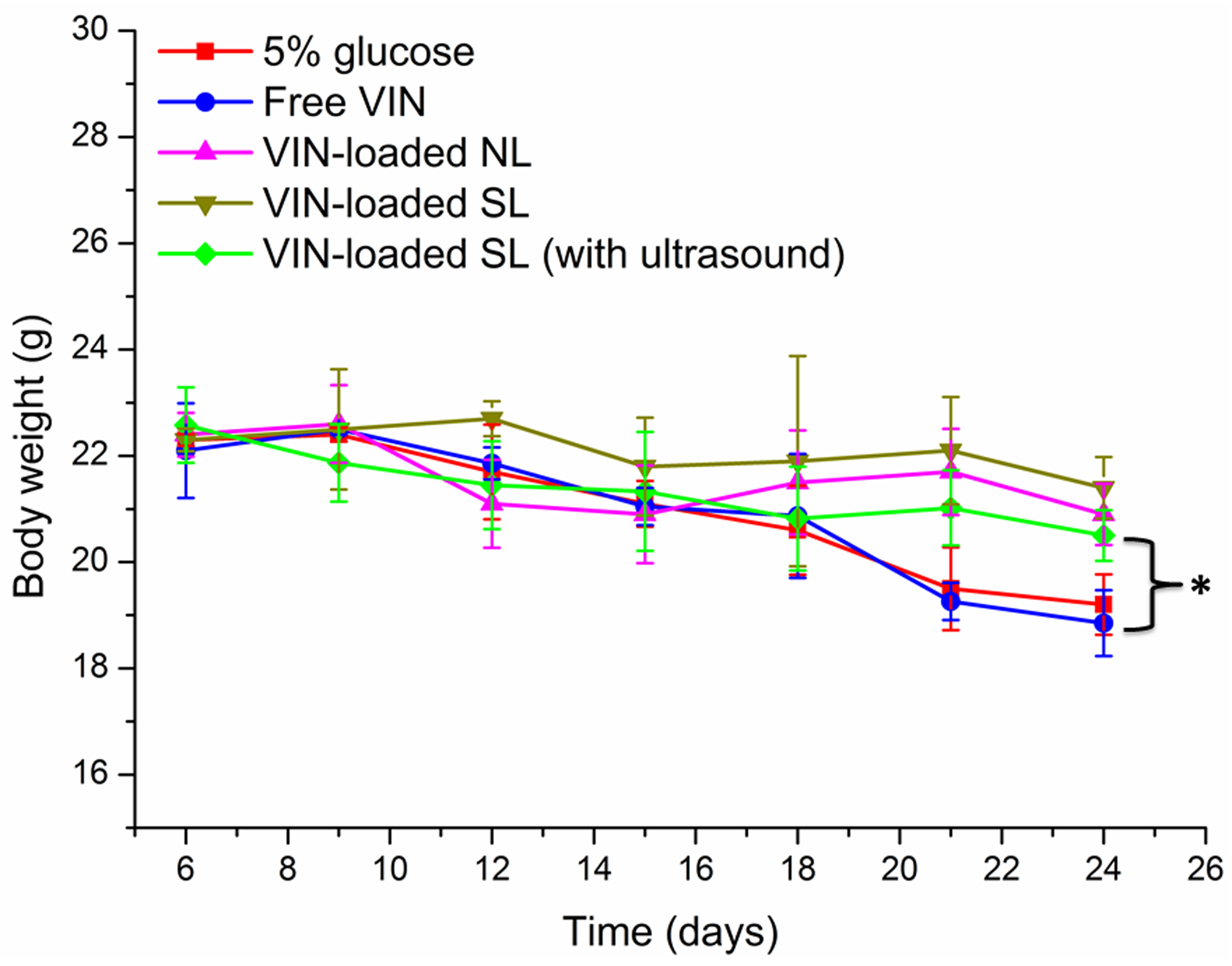

Supplement: Supplemental Material [file IDRD_A_1639845_SM9330.zip › Fig._S4.tif]
